# Phantom headache: pain-memory-emotion hypothesis for chronic daily headache?

**DOI:** 10.1007/s10194-011-0307-7

**Published:** 2011-04-09

**Authors:** Sanjay Prakash, Purva Golwala

**Affiliations:** 1 Department of Neurology, Medical College, SSG Hospital, O-19, Doctor’s Quarter, Jail Road, Baroda, Gujarat 390001 India; 2 Department of Medicine, Medical College, Baroda, Gujarat 390001 India

**Keywords:** Pain, Headache, Chronic daily headache, Dementia, Memory, Pain memory, Emotions

## Abstract

The neurobiology of chronic pain, including chronic daily headache (CDH) is not completely understood. “Pain memory” hypothesis is one of the mechanisms for phantom limb pain. We reviewed the literature to delineate a relation of “pain memory” for the development of CDH. There is a direct relation of pain to memory. Patients with poor memory have less chance to develop “pain memory”, hence less possibility to develop chronic pain. Progressive memory impairment may lead to decline in headache prevalence. A similar relation of pain is also noted with emotional or psychiatric symptoms. Literature review suggests that there is marked overlap in the neural network of pain to that of memory and emotions. We speculate that pain, memory, and emotions are interrelated in triangular pattern, and each of these three is related to other two in bidirectional pattern, i.e., stimulation of one of these will stimulate other symptoms/networks and vice versa (triangular theory for chronic pain). Longstanding or recurrent noxious stimuli will strengthen this interrelation, and this may be responsible for chronicity of pain. Reduction of both chronic pain and psychological symptoms by cognitive behavioral therapy or psychological interventions further suggests a bidirectional interrelation between pain and emotion. Longitudinal studies are warranted on the prevalence of headache and other painful conditions in patients with progressive memory impairment to delineate the relation of pain to memory. Interrelation of headache to emotional symptoms should also be explored.

## Introduction

The neurobiology of chronic daily headache (CDH) is incompletely understood. Various theories have been proposed to explain the cause of continuous pain. Both peripheral and central mechanisms have been suggested to explain the chronicity of headache disorders [1]. Sicuteri [2] suggested central disnociceptive theory to explain migraine headache. Nowadays, it has been suggested that chronic pain of any part of the body itself may be an independent disease entity and there may be some common neurobiological basis (especially central mechanisms) for the development of chronicity in all pain disorders [3].

Although the term “(quasi) phantom head pain” was coined by Sicuteri [4], the hypothesis of “pain memory” (a well-known mechanism for the development of phantom limb pain) has been suggested only quite recently in a few case reports of headache disorders. Prakash and Shah [5] reported three patients with hemicrania continua (HC) in whom complete response to indomethacin was noted a few months after the initiation of indomethacin therapy. The authors suggested the possibility of “pain memory” hypothesis for the delayed response to indomethacin. Recently, Marmura et al. [6] suggested the possibility of “pain memory” hypothesis for interictal pain in cluster headache (CH). Phantom pain is usually described in relation to surgical removal of the body parts such as limb, breast, teeth, etc. Harris [7] raised a question, “Is phantom pain a phenomenon only of phantom limbs or may it occur in people whose bodies are physically intact?” If “pain memory” can cause pain after amputation, it can be said that even a few episodes of pain in pre-amputated state may be because of “pain memory.” On this basis, we can speculate that chronic pain, including CDH, may be, at least in part, because of “pain memory.” Herein, we reviewed the literature to delineate a relation of “pain memory” to CDH.

## Phantom limb pain and chronic daily headache: any similarities?

Regarding phantom limb pain, the “pain memory” hypothesis was first postulated on the basis that the pain in the phantom limb is usually similar to the pain that existed in the limb prior to amputation. Several studies have confirmed that chronic pain prior to amputation is a powerful predictor for the development of phantom limb pain [8]. Minimal response of phantom limb pain to pre-emptive analgesia and marked improvement of pain following surgical removal of portion of SI cortex further strengthens the view of the “pain memory” hypothesis [8].

Chronic migraine (CM) and chronic tension-type headache (CTTH), two most common types of CDH, are usually transformed into the chronic form (from the episodic type) after months or years. Effective treatments of both CDH are challenging. CDH with daily pain is more treatment refractory than CDH without daily pain. CDH of longer duration is also known to have poor prognosis [9].

CH is usually considered as an episodic disorder. However, patients with chronic cluster headache (CCH) may have interictal pain [6]. The interictal pain was more in patients with long history of disease. The patients with interictal pain showed less response to therapies. The refractoriness was directly related to the duration of the interictal pain. Marmura et al. [4] suggested a possibility of “pain memory” for the interictal pain.

New daily persistent headache (NDPH) was initially considered as a benign disorder. In the first case series of NDPH, 68% male patients showed complete improvement at the end of 6 months [10]. However, recent reports considered it as the most refractory type of headache disorder. We reviewed the literature to look for the duration of headache at the time of presentation in various studies [11, 12]. Surprisingly, most of the patients in most of the studies had duration of headache of more than 6 months at the time presentation. Most of them had headache for greater than 1–2 years at the time of presentation. These indicate that most of the recent studies on patients with NDPH were done on those patients who already had headache duration of greater than 6 months. These may be the reason for not seeing the self-limiting form of NDPH. As patients with NDPH have almost daily or continuous headache since onset, these patients have a chance to develop “pain memory” in very early stage, and this may be the reason for refractoriness in NDPH. The drugs claimed to be effective in NDPH have shown effect only in the early stage of pain [13, 14].

Duration of pain (before amputation) is the most important predictor for the development of phantom limb pain. In the same line, recurrent headache of very long history is most important factor for the development of refractory headaches.

## Relation of pain with memory

It has been suggested that if memory of pain itself is a primary etiology of chronic pain, memory loss could result in pain reduction or pain-free state [15]. Therefore, it can be postulated that if patient already had memory impairment, there will be less chance to develop “memory” for painful conditions, and patients with memory impairment will have less chance to develop chronic pain.

### Pain in patients with dementia

It is believed that demented patients feel less pain or report less pain [16]. Persons with dementia are prescribed analgesics less frequently than are non-dementia persons [17]. However, a few recent observations suggest that a patient with dementia may have more acute pain than chronic form [18]. Although a few earlier studies have demonstrated increased tolerance for pain in demented patients, recent observations contradict this, and unchanged or even increased pain processing after painful stimuli have been observed in patients with dementia [19]. Similarly, Cole et al. [20] have demonstrated unchanged or increased brain responses to mechanical pressure in patients with AD.

As far as relation of headache with dementia is concerned, there are very few articles in the literature. Recently, a few observational studies have demonstrated increased prevalence of headache in pre-clinical state of familial AD [21]. Takeshima et al. [22] studied the prevalence of headache in subjects with dementia. They reported low prevalence of headache in dementia patients. The degree of dementia showed a significant relation with the headache; 30% of pre-dementia subjects and 16% of dementia subjects had headaches.

Trauma to the brain is known to cause chronic pain. Patients with mild traumatic brain injury (TBI) are known to have higher prevalence of chronic pain than those with moderate to severe TBI [23]. In a recent review [23], the prevalence of chronic pain following mild TBI was significantly greater in comparison to that following moderate or severe TBI (75.3 vs. 32.1%). In a retrospective study [24], posttraumatic CDH was noted in 80% with mild TBI. Conversely, only 27% patients with moderate to severe head injury had CDH. In another study, Formisano et al. [25] examined the 500 patients suffering from very severe TBI. The incidence of headache was only 10%. Posttraumatic headaches were more in patients who made good cognitive recovery. The prevalence of posttraumatic CDH is higher among people who have a history of recurrent headaches in the past [26]. This may be because of ‘pre-existing’ or ‘early’ or ‘marked’ “pain memory” because of previous recurrent headaches.

Taken together, it can be said that memory modifies the frequency, duration, and intensity of repeated noxious stimuli and “impaired memory” will prevent the formation of “pain memory.”

## Common neurosignature for chronic pain?

Recently, various structural abnormalities have been reported in patients with chronic pain, including phantom pain and headache [27]. The changes in gray matter in chronic pain patients were noted mainly in structures involved in pain processing. The morphological changes noted in headache disorders have been replicated in most of the studies investigating brain changes in different types of chronic pain. The common neurosignature suggests common mechanisms for chronic pain, and mechanisms responsible for the development of phantom limb pain may also have a role in CDH and other chronic painful conditions. A few authors have suggested that cortical reorganization in the brain may play a part in the development of “pain memory” and in the persistence of pain [28].

## Common neural network for pain and memory?

Apkarian et al. [29] performed a meta-analysis to identify the structures involved in pain processing. The main structures involved in processing for acute pain are primary and secondary somatosensory, insular, anterior cingulate, and prefrontal cortices and thalamus. Other less commonly involved areas are basal ganglia, cerebellum, amygdalae, hippocampus, and areas within the parietal and temporal cortices. The involvement of these structures depends on various factors, especially type of stimuli and chronicity of pain.

Svoboda et al. [30] performed a meta-analysis to identify the structures involved in memory recollection. The ‘core’ network for memory includes medial and ventrolateral prefrontal, medial, and lateral temporal and retrosplenial/posterior cingulated cortices, the temporoparietal junctions, and the cerebellum. Other less commonly involved areas are dorsolateral prefrontal cortex, superior medial and superior lateral cortex, anterior cingulated, medial orbitofrontal, temporopolar and occipital cortices, thalamus, amygdalae, etc.

Review of the literature suggests that a larger number of structures are common to both (pain and memory) networks. All six major structures of pain matrix are also a part of the memory network. Most of the structures, noted as abnormal in neuroimaging in chronic painful conditions, including CDH, are also the part of memory network. Prefrontal cortex (PFC) was noted as a major structure involved in both the pain and memory networks. SI is the main site for pain perception. However, SI may be involved even in memory formation. Pavlovian conditioning, a form of associative learning, is thought to play an important role in the acquisition and exacerbations of pain-related responses. Various data suggest that SI contributes to maintain a memory trace in Pavlovian conditioning, and a possibility of a close relationship between perceptual pain and remote memory process exists [31].

The hippocampus is widely considered as an organ for memory and emotion. However, recently, a few neuroimaging studies on various chronic painful conditions have demonstrated structural changes in the hippocampus. These observations hint a role of hippocampus in patients with chronic pain and a possible interrelation between pain, memory, and emotions.

Furthermore, the limbic system network overlaps that of pain and memory [32]. Prefrontal, cingular and insular cortices (components of the limbic system) are activated during the majority of neuroimaging studies of pain and memory, and these areas have been implicated in the affective processing of pain and memory. In addition, components of the limbic system, such as amygdalae, have been implicated in persistent pain.

## Pain-memory-emotion hypothesis (triangular theory) for chronic pain?

**Fig. 1:**
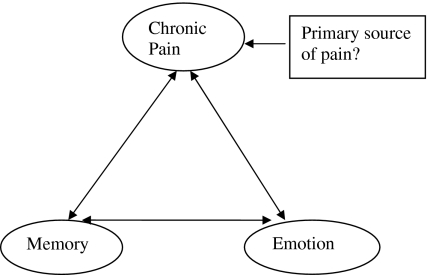
Relation of chronic pain to memory and emotion

Review of the literature suggests a marked overlap between neural network for pain, memory, and emotion [29, 30, 32]. All these networks are predominantly localized to the dominant (left) side of the brain, further suggesting close interrelations among these three. We speculate that these structures are interrelated in a triangular fashion, and each of these is related to the other two in a bidirectional pattern, i.e., stimulation of one of these networks may excite the other (Fig. 1).

### Relation of pain to emotion

Patients with chronic pain are known to have increased prevalence of emotional/psychiatric abnormalities. The prevalence of psychiatric disorders is highest in patients with CDH than in patients with less frequent headache; hence the interrelation between the pain network and the limbic system [33]. Observational studies have demonstrated that stress may both precede and follow the onset of headache. This reciprocal relation between stress and headache indirectly hints that excitation of limbic system will stimulate the pain network and vice versa [34]. Chronic pain may be triggered by observing, or inferring that another person is in pain, indicating stimulation of pain network by limbic system. Hypervigilance in the limbic system is also known to increase pain-processing activity in the central nervous system, and limbic system hypervigilance has been suggested as a contributing factor for chronic pain. [35]. Feeling of anxiety, fear, etc., may also aggravate or induce chronic pain. Pain-related fear has been suggested as a risk factor for the development and persistence of chronic pain [36].

Cognitive behavioral therapy is known to reduce both chronic pain and psychological dysfunctions [37]. The improvement of chronic pain, including CDH, usually parallels with the improvement of psychiatric abnormalities [33]. In a few studies, these improvements were accompanied by reduced limbic activity [37]. Recently, Shaw et al. [38] suggest that lifetime history of depression or anxiety may be risk factors for the transition to chronicity in men with first-onset LBP. This suggests that ‘pre-existing’ emotional abnormalities in patients with acute pain may lead to early strengthening of the triangle and therefore, more chance for the transition to chronicity. These all indirectly hint that both emotions and pain are interrelated in bidirectional pattern.

### Relation of pain to memory

Apkarian et al. [39] suggested that pain and learning and memory are intimately related. Memory is often retrieved involuntarily. However, it can be retrieved voluntarily by just thinking about the pain. In Giummorra et al. [40] case series, phantom limb pain was triggered by just thinking about the pain. In the same line, background headache was aggravated by thinking of pain in patients with HC [5]. As thinking of pain may exacerbate or induce pain, fear to get severe headache may stimulate memory and/or emotional network, which will ultimately stimulate the pain network. In many primary headache disorders, acute attacks are very severe and patients remain fearful for headache induction. A few studies have demonstrated that patients may feel pain even on hearing the words having meaning of pain [28].

### Relation of memory to emotion

Emotions and memory are closely related to each other. Emotion of an event can exert effects at the time of encoding, during retrieval as well as during the experience of recollection. Events with emotional components are easily retrieved. Emotional intensity also affects the properties of memories, such as degree to which memory is re-lived and the vividness of the memory [41]. Pavlovian conditioning also strengthens the view that emotion and memory are intimately related. The mechanisms of Pavlovian conditioning have also been used to explain the mechanisms of chronic pain [31].

## Clinical implications

**Fig. 2:**
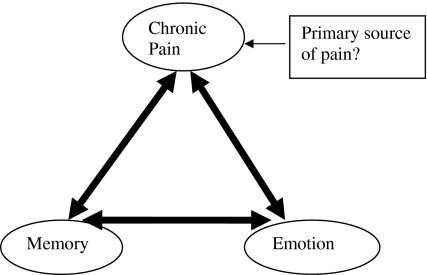
Strengthening of interrelation between pain, emotion, and memory (with progression of time)

Chronic pain, memory, and emotions could be interrelated in a bidirectional pattern. Stimulation of any one may excite others and a vicious circle may form, and it will progressively strengthen the interrelation. Continuous ongoing noxious stimuli (responsible for beginning of interrelation) will also continue to strengthen the interrelation (Fig. 2).

Chronic pain produces long-lasting structural abnormalities. Even emotional or behavioral abnormalities are known to produce long-lasting structural changes in the brain [42]. Consolidation of memory is also a longstanding process [43]. These all may be the reasons for refractoriness of chronic pain.

Memory theories propose that memories are stable once stored. Older memories are more resilient to damage than recent memories. Therefore, chronic pain ought to be prevented as early as possible in order to keep “pain memory” from being established. However, recently various methods or paradigms have been developed for suppressing unwanted memories [44, 45]. These paradigms can be used to suppress ‘pain memories.’ The pain experience also depends on attention. It is perceived as less intense when somebody is distracted. Conversely, it increases when attention is focused on pain [41]. This principle can be used to prevent the further strengthening of “pain memories.”

As person may have conscious control over emotions, it may be the weakest point of triangle and interventions on this part may be relatively easy. Cognitive therapy may help in preventing exacerbation/induction of pain and further formation of pain memories.

Morphological changes observed in various painful conditions may be corrected if pain (including primary source of pain) is treated completely [46]. However, most of the chronic pain conditions, including CDH, have not obvious primary source of pain. Therefore, primary pathophysiology responsible for the pain (i.e., pathophysiology responsible for generation of pain in early stage) will continue even in chronic form. It will continue to strengthen the relation of pain to emotion and memory. Therefore, primary aim should be to prevent the progression of recurrent acute pain into chronic form. However, in well-established chronic pain, multiple interdisciplinary interventions may be required with targeting each component of the triangle responsible for the generation of chronic pain.

## Conclusion

Pain, memory, and emotions are closely interrelated. The interrelation gets stronger with increasing duration (i.e., chronicity). Longitudinal studies are warranted on the prevalence of headache and other painful conditions in patients with progressive memory impairment to delineate the relation of pain to memory. Studies on temporal relation of exacerbation of headache to emotional features are also required to delineate the bidirectional relation between pain and emotion.
